# Use of Botulinum Neurotoxin in Parkinson’s Disease: A Critical Appraisal

**DOI:** 10.3390/toxins13020087

**Published:** 2021-01-25

**Authors:** Wolfgang H. Jost

**Affiliations:** Parkinson-Klinik Ortenau, Kreuzbergstr. 12, 77709 Wolfach, Germany; w.jost@parkinson-klinik.de; Tel.: +49-7834-971212; Fax: +49-7834-971340

**Keywords:** botulinum neurotoxin, Parkinson’s disease, sialorrhea, blepharospasm, torticollis, tremor, focal dystonia, dyskinesia, camptocormia, Pisa syndrome, non-motor symptoms

## Abstract

For well over 30 years, the botulinum neurotoxin (BoNT) has been used for a large number of indications, some of which however have not been licensed. Admittedly, approval varies in many countries and this permits a large spectrum for evaluation. Thus, BoNT is used for patients with Parkinson’s disease (PD) and other Parkinson’s syndromes (PS) in varying degrees of frequency. We have to distinguish between (1) indications that are either approved or (2) those not approved, (3) indications that might be a result of PS and (4) finally those which appear independent of PS. The most important indication for BoNT in PS patients is probably sialorrhea, for which approval has been granted in the majority of countries. Cervical dystonia is a frequent symptom in PS, with anterocollis as a specific entity. A further indication is blepharospasm in the different forms, especially the inhibition of eyelid opening in atypical PS. The use of BoNT in cases of camptocormia, the Pisa syndrome and neck rigidity is still a matter of debate. In dystonia of the extremities BoNT can be recommended, especially in dystonia of the feet. One well-known indication, for which however sufficient data are still lacking, involves treating tremor with BoNT. As to autonomic symptoms: Focal hyperhidrosis and detrusor hyperactivity can be mentioned, in this last case BoNT has already been approved. A number of further but rare indications such as freezing-of-gait, dyskinesia, and dysphagia will be discussed and evaluated.

## 1. Introduction

For over 30 years, botulinum neurotoxin (BoNT) has been used for numerous indications, some with level A recommendations [1,2,3,4]. In the meantime, many indications has been approved, whereby considerable differences persist from country to country. In the majority of countries the following indications have been approved ([5]; SPC Botox^®^, SPC Dysport^®^, SPC Xeomin^®^):

torticollis spasmodicus (cervical dystonia)
blepharospasm
hemifacial spasm
spastic equinus in cerebral palsy
focal spasticity (arm and foot)
axillary hyperhidrosis
chronic migraine
neurogenic/idiopathic bladder
sialorrhea
spasmodic dysphonia (approved only in some countries).

Considering the use of BoNT in Parkinson’s syndrome (PS), we can distinguish between: (1) uses in indications independent of the underlying disease (co-morbidities); (2) indications induced by the primary disease and of course (3) indications which represent symptoms of PS. Within symptoms of PS, we can further distinguish between motor und non-motor ones [6]. In this review, only those symptoms will be treated which are directly associated with the underlying disease PS itself. Then the distinction has to be made between (1) approved indications and (2) those which have no approval and finally (3) additional indications for which BoNT at present only finds experimental use (Table 1). It is interesting to note that some indications have been formulated so strictly that the specific indication in PS does not conform to the approval. 

## 2. Approved Resp. Licensed Indications

### 2.1. Blepharospasm

In blepharospasm, we can distinguish between an orbital and a palpebral type [3]. A specific variant is the so-called eyelid-opening inhibition type or involuntary levator palpabrae inhibition [3]. This form in turn has to be distinguished from apraxia of lid-opening. These two terms are frequently used interchangeably [4,7,8]. Strictly speaking, this is not an example of apraxia, which means that the specific form of apraxia requires separate treatment, without being an indication for BoNT. Of course, every form of blepharospasm can occur in PS. Qualitative data on the frequency are not available—only case studies have been published to date [3,4]. Here we have to discuss whether blepharospasm in Parkinson’s patients is seen as primary or secondary, although this differentiation does not appear to have influence on the therapeutic success [9].

Blepharospasm of the eyelid-opening inhibition type frequently occurs in cases of atypical PS (APS), but also in Parkinson’s disease (PD). According to Lepore and coworkers it is characterized by transient inability to initiate lid opening, no overt orbicularis oculi spasm, vigorous frontalis contractions, and absence of oculomotor or ocular sympathetic nerve dysfunction or ocular myopathy [8].

**Figure 1:**
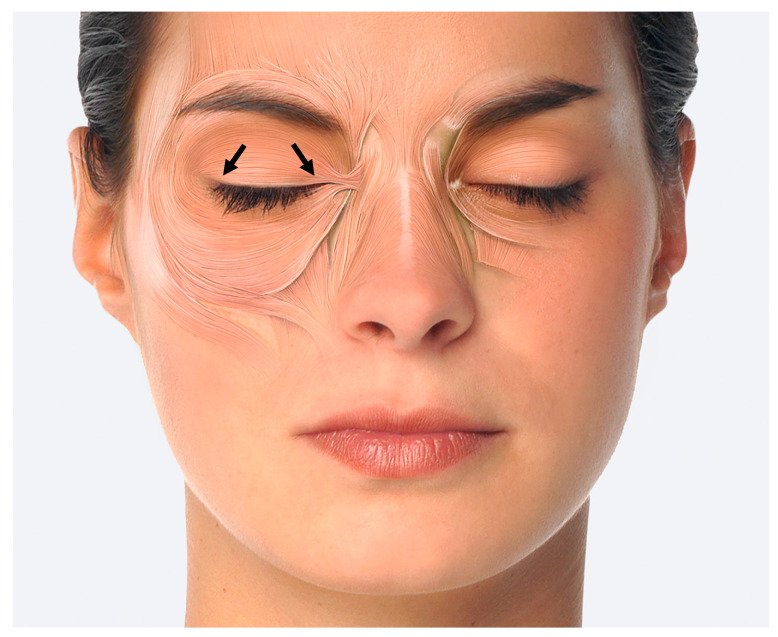
Tarsal injection of the orbicularis oculi muscle [14].

Differential diagnosis for these various forms can be achieved with electromyography (EMG) [8]. Diverging from the standard injection plan, an injection close to the eyelid in the tarsal upper lid is recommended for therapy in cases of the eyelid-opening inhibition type (see Figure 1) [3,10,11]. Up to now, little data have been published on the success of this therapy. Overall the therapeutic success is rated as possible, but as less effective than in the orbital and palpebral types [10].

In 1995, Lepore et al. already published a larger collection of case studies describing good success [11]. In a further study with 12 patients, further good results could be documented for pretarsal injection, although the study does not detail whether any one of the patients suffered from a PS. Likewise, the exact dose is not given [12]. A further study reports on two PSP (progressive supranuclear palsy) patients with apraxia of eyelid opening in whom an injection toward the junction of the preseptal and pretarsal parts of the palpebral orbicularis oculi muscle improved eyelid motility [13].

This apraxia also occurs frequently in patients with APS, but in these cases, therapy with BoNT is not indicated. The question is still subject to debate as to whether this is apraxia in the narrow sense or rather involves the eyelid-opening inhibition type [8]

Clinical consequence: In blepharospasm, the injection of BoNT can be viewed as the therapy of choice. Injecting is done in the routine way. In the eyelid-opening inhibition type a therapeutic trial is well justified, whereby an injection in the tarsal section of the orbicularis oculi muscle is recommended, and where required, with a somewhat higher dose than usual, for example 5 U inco-/onaBoNT/A or 20 U aboBoNT/A per site.

### 2.2. Cervical Dystonia

In PS, cervical dystonia (CD) frequently occurs, which is usually treated exactly as spasmodic torticollis. Concrete data on the frequency of CD in PS are not available—there are merely smaller reports and single case studies. Thus, for example, in one study on 74 PD patients, 33.9% presented with CD [15]. In addition, anterocollis was found in six resp. 9.4% [15,16]. Our clinical experience considers rotatory torticollis less frequent and anterocollis more frequent than in CD [3,16].

**Figure 2:**
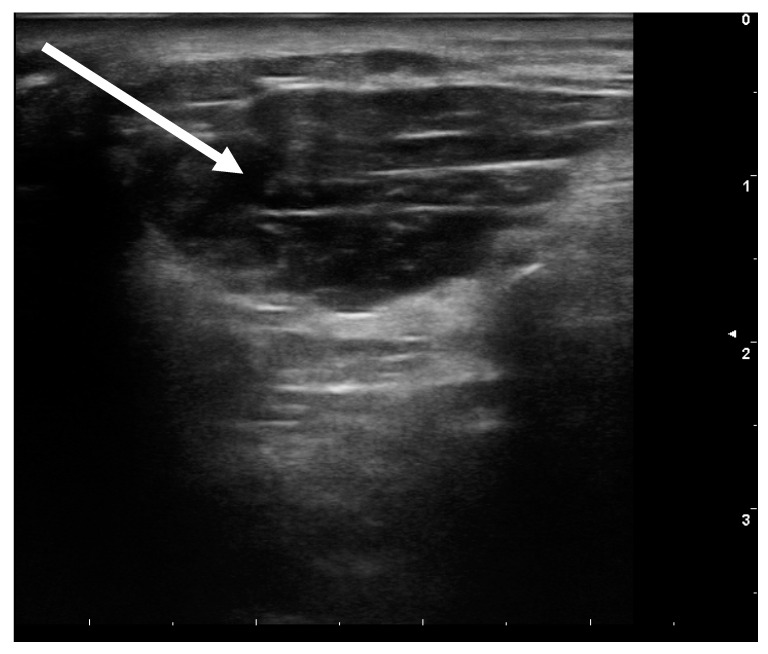
Ultrasound image of the levator scapulae muscle. Clearly visible are the four parts of the muscle and the change in morphology compared to the norm (notably rounded).

In anterocollis, we have to distinguish whether it is a dystonic activation of the ventral musculature or whether proper differential diagnostic work would reveal merely a weakness in the neck extensors (so-called dropped head). The dystonic form can be viewed as basically similar to camptocormia (see Section 3.2). A clinical examination usually suffices for making the differential diagnosis, in individual cases guided by EMG [16,17]. A useful test consists of having the patients lift their arms up: if raising the head is then facilitated, essentially this would indicate that the levator scapulae muscle is involved, a muscle that is quite easily injected [17]. Under ultrasonic control (see Figure 2) BoNT is injected with a usual dose, i.e., 50 U ona-resp. incoBoNT/A or 150 U aboBoNT/A, the effect becomes apparent after a few days and persists for approximately three months. This injection can be repeated regularly. Due to our experience a moderate improvement can be established in many cases, and in some cases an adequate lifting of the head is possible. Controlled studies, however, are not yet available, an RCT is presently being conducted. If the ventral neck muscles are involved, then diagnosis and injecting are difficult [17], while if a weakness in the neck extensors is the case, injecting with BoNT should be avoided. Neck rigidity is very often found in the PSP [3]. The use of BoNT is also discussed here, no final recommendation can be made.

Clinical consequence: In cases of CD, injection with BoNT may well be seen as the therapy of choice, and this holds true of course for CD in PS, whereby the injection is conducted according to routine protocols. A distinctive case is found in anterocollis, in which an examination for each individual patient must be made to establish whether dystonia or muscle weakness pertains. Of course, in the case of muscle weakness, an injection would be rejected. Anterocollis is more difficult to treat than the other forms of CD [17].

### 2.3. Siallorhea

The most important indication for BoNT in PS is sialorrhea, or drooling [18]. PD patients do not actively produce more saliva, instead they fail to swallow their saliva sufficiently (impaired oropharyngeal swallowing. inadequate clearance, with anterior and posterior pooling), for which reason saliva is repeatedly released [18]. This is reinforced through forward head posture as well as incomplete mouth closure. Medication for reducing saliva production typically has systemic side effects [18]. For a number of years, BoNT/A and BoNT/B have proven useful here. At present, there is qualitative data for abo-, inco-, onaBoNT/A, and rimaBoNT/B [18,19,20,21,22,23,24,25,26,27,28].

In 2019, the FDA (US Food and Drug Administration) approved incoBoNT/A and rimaBoNT/B for chronic sialorrhea in adults [SPC Xeomin^®^]. The groundwork for approval was the so-called SIAXI study, a large controlled study that confirms the efficacy and safety of incoBoNT/A for the treatment of sialorrhea due to PD and APD [29]. The study demonstrated a dose-dependent efficacy with reduction in uSFR (unstimulated salivary flow rate) and improvement in the GICS (Global Impression of Change Scale) compared with a placebo for four weeks post-treatment and reached statistical significance for 100 U incoBoNT/A; the efficacy was confirmed by secondary and other endpoints. The reduction in uSFR and improvement in drooling measured by GICS and by DSFS (Drooling Severity and Frequency Scale) were maintained at week 16, indicating a duration of efficacy and benefit to the patient beyond four months after the initial injection. IncoBoNT/A 75 U dose was more efficacious than placebo, but the efficacy was inferior in comparison to 100 U. In the extension period mean uSFR decreased consistently with repeated injections [30].

In the approval study with rimaBoNT/B 122 PD patients (out of a total of 187 patients) were randomized to 2500 U, 3500 U rimaBoNT/B or placebo. Treatment with both doses significantly reduced uSFR at week four vs. placebo. The CGI-C scores were also significantly improved at week four for both treatment groups vs. placebo. Treatment benefits were seen as early as one week after injection and were maintained over the treatment cycle of approximately 13 weeks. The most common adverse events were mild to moderate dry mouth, dysphagia, and dental caries [31].

In the meantime, BoNT has been approved for this indication in many further countries. The recommended dose is a total of 100 units (U) incoBoNT/A, 60% parotid gland, and 40% submandibular gland, for rimaBoNT/B 500–1500 U per parotid gland and 250 U per submandibular gland (total dose 1500 to 3500 U). As frequent side effects dry mouth and dysphagia were detailed [18,29,30,31].

Clinical consequence: Sialorrhea injections of BoNT are now seen as the therapy of choice, and this holds for PD and APS patients. BoNT is safe and efficacious.

**Figure 3:**
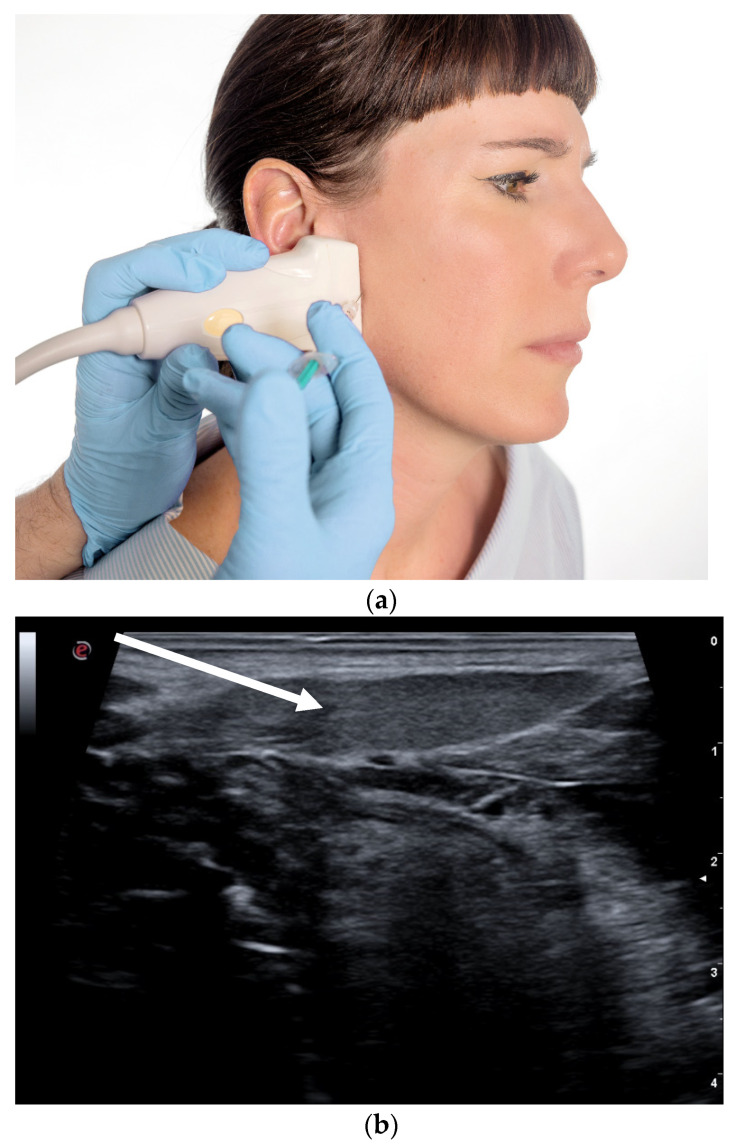
(**a**) Injection into the parotid gland; (**b**) sonography of submandibular gland.

The injection is conducted under ultrasonic control (see Figure 3) into the salivary glands, which can reduce salivary production for several months [18]. Usually injections are made into two salivary glands on each side (parotis and submandibular gland). Local bleeding and dry mouth may occur, and the effect diminishes completely with time.

### 2.4. Detrusor-Hyperactivity (Overactive Bladder)

Parkinson patients, both PD and APS, present with a preponderance for an overactive bladder (OAB) [32,33,34,35,36,37]. The typical anticholinergic or antimuscarinergic therapy is not sufficient or has strong side effects. In these cases, the endoscopic injection of BoNT into the detrusor can be of service [32,33]. OnaBoNT/A gained approval for the treatment of OAB arising from an underlying neurological cause. Usually, 100 U of onaBoNT/A (up to a maximum of 300 U) are injected, but with the risk of inducing postvoid residual urine. Unfortunately, the data available on PD is quite limited, as only smaller studies have been published [33].

Giannantoni and coworkers demonstrated [34] that a detrusor injection of 100 U onaBoNT/A induced clinical and urodynamic improvement in OAB symptoms that lasted at least six months in patients with PD. Kulaksizoglu et al. reported good success for up to nine months in 16 PD patients who had been treated with 500 U aboBoNT/A [35]. In another study with 20 patients resistant to antimuscarinergics, 100 U of onaBoNT/A were successful (50% incontinence decrease) in 59% with moderate to marked improvement [36].

Improvement was described in 19 of 24 (79.2%) patients with OAB in an open label, retrospective study [37]. A total dose of 100 U onaBoNT/A was used. In seven patients (29.1%) with complete resolution of urgency urinary incontinence. Out of 49 injections in total, only five caused incomplete bladder emptying requiring the use of intermittent catheterization (10.2%).

Clinical consequence: In cases of detrusor overactivity or OAB, injection with BoNT can be taken into consideration to treat extensive symptoms when oral medication proves insufficient. This therapy is traditionally conducted by urologists under endoscopic control. In PD patients lower doses are recommended (e.g., 100 U onaBoNT/A).

## 3. Non-Licensed Indications

### 3.1. Tremor

BoNT is indicated for muscular overactivity, and for this reason its use for tremor and in particular for Parkinson-tremor suggested itself early on [3]. Furthermore, tremor as a symptom in PD reacts poorly to the standard approved medication [38]. PD patients can present different types of tremor: resting, as well as postural and kinetic tremor [39]. And thus a distinction has to be made as to just which form of tremor is to be treated.

Unfortunately the previous results published to date were not convincing [39,40,41,42,43]. With new techniques and the use of smaller doses the chances of success were improved [44]. Relevant studies are being conducted at present.

A recent study that treated 30 PD patients with incoBoNT/A injected into the hand and forearm muscles revealed a statistically significant improvement in clinical rating scores of rest tremor and tremor severity, with no relevant difference in grip strength [45].

Samotus and coworkers [44] injected a mean initial dose of 174.1 ± 68.8 U incoBoNT/A in 8.4 ± 1 muscles. Fourteen percent (in four out of 28 patients) withdrew due to bothersome hand weakness but had some tremor improvement, 11% (3 of 28) withdrew due to lack of benefit from treatment but did not have any weakness (12 withdraw altogether). Kinematics detected a significant reduction in PD tremor amplitudes by 70% over the treatment course. In an open study the same study group found a significant improvement of tremor amplitude in 41.6%, and improved quality of life in 23%, with efficacy in 45% of participants [46].

Improved efficacy was demonstrated for essential tremors [47,48,49], but this was not specific for PD patients.

In addition to tremors of the extremities, PD patients frequently have a tremor affecting chin, lips, jaw, and tongue. Jaw tremor was beneficially treated with injections of BoNT into the masseter muscles in three PD-patients [50].

Clinical consequence: At present, the injection of BoNT cannot be recommended for treatment of tremors in PD. We have to await the results of studies that are presently being conducted, but in individual cases, injection may well be worth taking into consideration The possibility of a functional paresis is a limiting factor [51], thus making a personalized approach advisable [46].

### 3.2. Camptocormia and Pisa syndrome

Anterocollis (see Section 2.2), camptocormia and the Pisa syndrome can be seen as postural deformities [52].

The pathogenesis of camptocormia is still a matter of controversy and the exact pathophysiology is not fully understand [52]. Camptocormia is often a very disabling condition, entailing an extreme anteflexion of posture, often combined with lateral bending (all the way up to a Pisa syndrome), and often worsen with ambulation. This often makes normal standing and sitting almost impossible and the patients frequently become wheel-chair bound [52]. In cases in which dystonia presents with increased muscle tonus, the musculature of the abdominal wall and the hip flexor can become involved as well [52,53,54,55]. Therapy presents considerable difficulties and therapeutic success is limited. Use of BoNT should only be considered as an option in cases with unequivocal dystonia.

Several studies on injections into the iliopsoas muscle proved negative [53,54,55], likewise with injection targets into the psoas [56]. In an early study, four of nine patients showed improvement after BoNT injections into the rectus abdominus muscles [57]. The authors are surely correct in concluding that this heterogeneous disorder has multiple etiologies.

Data on success in using BoNT in Pisa syndrome, as a form of lateral trunk flexion, are even less satisfactory. In such cases, an overlapping of the two symptoms occurs frequently in the patient populations because many patients with Pisa syndrome present with camptocormia and vice versa.

In a study by Bonanni et al., nine patients with lateroflexion (Pisa syndrome) were injected with BoNT and placebo, in a blinded cross-over (after three months) study design. Under EMG control, the patients received 4 × 125 (1 mL each) units aboBoNT/A into the paraspinosus muscle. All patients were videotaped and evaluated with the Trunk Dystonia Disability Scale (TDDS), a Visual Analogue Scale (VAS) and a goniometric measurement of the lateral displacement. In the placebo group no benefit was registered, but in the BoNT-group, treatment was effective in six patients. Four patients have had ongoing BoNT treatment for the two years since the study [58].

In the subsequent study, an RCT performed by Tassorelli and coworkers [59], 26 patients were tested either with BoNT (*n* = 13) or placebo (*n* = 13), while both groups received intensive physical therapy. The precise sites of injection were determined for all patients according to their EMG report and individual injections were permitted. The iIliopsoas muscle alone was injected in all the patients. The total dose ranged between 50 and 175 U incoBoNT/A. The verum group benefited in particular in measures of their pain but also showed a more prolonged efficacy on several clinical and kinematic variables. It is interesting to note that both groups improved significantly in terms of static postural alignement and in range of motion [59].

In a case report, good success was reported after injection of 50 U incoBoNTA into the quadratus lumborum [60].

In a small prospective study, six patients with PD and camptocormia were treated with 75 or 90 U onaBoNT/A, with injections into each external oblique muscle bilaterally under sonographic guidance. The mean camptocormia angle showed significant attenuation (38° vs. 18°) [61], whereas a degree of flexion of at least 45° of the thoracolumbar spine is mostly used to define camptocormia [62]. Subjective relief was present in four cases [61].

Another pilot study with 15 patients evaluated the efficacy of BoNT injection in paraspinal and non-paraspinal axial muscles after magnetic resonance imaging and sonographic-guided electromyography evaluation [63]. A beneficial response was obtained in 11 of 13 patients (84.6%), with 40% average reduction in trunk bending, and 52.2% amelioration of pain/discomfort at the Visual Analogue Scale (VAS).

Clinical consequence: In recent years, BoNT has been applied in different patients for treating camptocormia and/or a Pisa syndrome. EMG and/or sonography has been performed in all patients beforehand, with injections into the psoas, paraspinal musculature and that of the abdominal wall, depending on the clinical findings. Unfortunately, up to the present, only temporary and slight, and non-lasting success has been achieved. But in individual cases, a therapeutic trial can well be indicated [64].

### 3.3. Focal Dystonia

Dystonia is common in PD patients, especially in younger patients, occasionally as a primary symptom, more frequently as a motor complication of levodopa therapy [38,65]. Descriptions on laryngeal dystonia, oromandibular dystonia, bruxism, and writer’s cramp were published. The patients should be treated in the same way as usual [65]. Other features are, e.g., striatal hand and more often, striatal toe [66].

#### 3.3.1. Laryngeal and Oromandibular Dystonia

Spasmodic dysphonia can occur in all Parkinsonian patients, in both the abductor and the adductor subtype [67]. BoNT is the therapy of choice and even approved in some countries [1]. Unfortunately, there have not yet been any specific studies on the use of BoNT for cases of laryngeal dystonia for PS [67].

The same holds true for oromandibular dystonia [1]. There is good evidence for successfully using BoNT here, but not for its specific use in PS.

#### 3.3.2. Dystonia of the Upper Limbs

Focal dystonia of the upper limbs is often task-specific. For example, writer’s cramp is frequent, and striatal hand as well [65,66]. While walking, a dystonic movement of the arm frequently occurs with malpositioning of the hand. There are various studies on this, though unfortunately none on PD patients [1].

A small study on treatment for “dystonic clenched fist” in three CBD (corticobasal degeneration), and seven PD patients, examined the effect of BoNT. All patients had some degree of flexion at the proximal metacarpophalangeal joints and required injections into the lumbricals. The effect depends on the severity of the deformity and the degree of contracture. All patients had significant benefit to pain, with accompanying muscle relaxation, and palmar infection, when present, was eradicated. Four PD patients obtained functional benefit [68].

#### 3.3.3. Foot Dystonia

**Figure 4:**
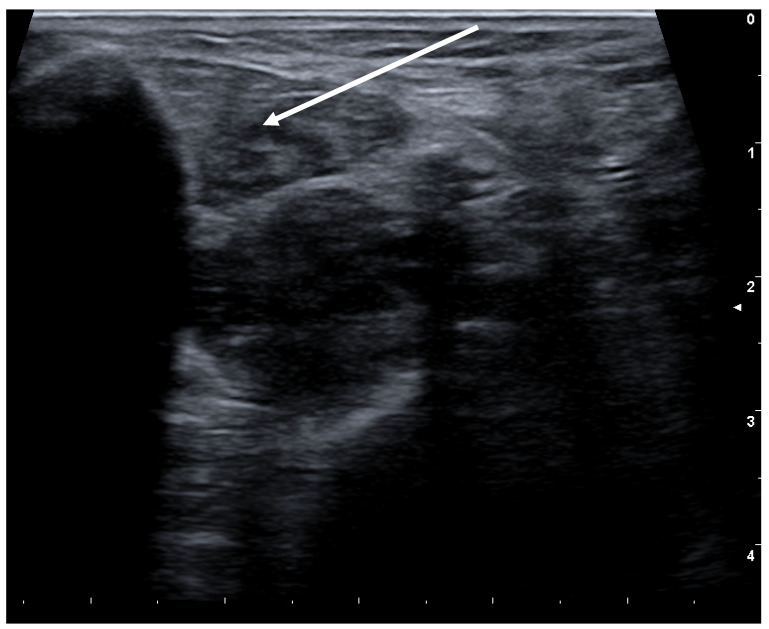
Sonography of the calf with the long toe flexor (M. flexor digitorum longus).

Parkinson patients frequently have dystonia in the feet: kinesigenic and exercise induced foot dystonia, considered a hallmark of early-onset PD [65]. Most frequently this involves foot dystonia as an off-phenomenon [65] and may occur over the full course of a day, but is more frequent in the early morning hours. In addition to functional disability the patients complain most particularly about their having pain (see below). When these symptoms are dependent on the dopaminergic stimulation adapting the medication accordingly is recommended. But usually the involuntary movements prove independent of the dosage. In these cases BoNT can be used. The major forms of foot dystonia include:

(1) Involuntary extension of the big toe:
  The other toes remain in the resting position or move only slightly, over-flexing takes place in the big toes [66]. Therapy with BoNT has long been well established for these cases [69]. Here an injection into the extensor of the big toe (Musculus extensor hallucis longus). This muscle is positioned in the lower calf; a single injection suffices.
(2) Flexion of the toes:
  This second form usually causes more discomfort to the patients, as it induces involuntary movements in the toes, in particular the small ones, but also the big toes. It occurs frequently as exercise-induced dystonia. Here, a distinction has to be made whether only one of the two or both toe flexing muscles are involved. The short flexor is found in the sole of the foot, the long flexor in the lower calf. Here too the injection into the muscle is performed under ultrasonic guidance (see Figure 4).
(3) Inversion and/or supination of the foot:
  This form of dystonia is frequently more complex and difficult to treat, as it causes occasional functional paralysis with an increased risk of falls resulting. A therapeutic trial with low dosage may be indicated in individual cases.

In an open-label study with BoNT, Pachetti and coworkers [70] reported on 30 PD patients with painful foot dystonia all of whom benefited from the therapy. In a controlled study, 45 PD patients with dystonic plantar flexion of their toes were treated with 100 U incoBoNT/A, or with placebo either in the flexor digitorum longus or the flexor digitorum brevis. The primary endpoint was the Clinical Global Impression (CGI) of change. Mean CGI was improved in the BoNT group compared to the placebo group (*p* = 0.039), with no difference of efficacy between the two injection sites [71]. In a further study on BoNT treatment in the specific muscles involved, a positive effect on pain and mobility was found in patients who had had deep brain stimulation (DBS) and developed foot dystonia [72]. The same group published results on improved walking in foot dystonia after BoNT injection [73].

#### 3.3.4. Dyskinesia

The use of BoNT has also been discussed for dyskinesia. Positive reports, however, are only found in single case studies [3,74]. In one publication by Espay et al., a small collective of patients with cervical dyskinesia was studied, but no positive relevant effect could be seen, instead only relevant side effects [74].

Clinical consequence: Although no approvals are yet available, the use of BoNT for focal dystonia in PD is a viable option. In fact, treatment of foot dystonia is the therapy with the best evidence. At present, there is only insufficient evidence for therapy of dyskinesias with BoNT. Focal dystonias are also common in CBD unfortunately there are no studies on this.

### 3.4. Freezing of Gait and RLS

Freezing of gait (FOG) is quite frequent in PS, especially in the advanced stage and unfortunately responds but poorly to medication [38]. For a number of years now the use of BoNT in FOG has been subject to study. The results, however, are not unequivocal [75,76].

In 1997, Giladi et al. described positive effects, which they then confirmed in a second study in 2001 [75,76]. BoNT/A was injected into the calf muscles of 10 PD patients: Seven reported improvement of FOG severity in 15 out of 17 therapeutic sessions. Four patients (40%) reported marked improvement in five sessions.

In a further study from the same study group, 150 U onaBoNT/A (n = 6) or normal saline (n = 5) was injected into each leg’s calf muscles. No improvement was observed in either group, but leg weakness and falls led to early termination [77].

Vastik and coworkers investigated 20 PD-patients with FOG. 10 patients with FOG were treated with BoNT injection into both tensor fasciae latae muscles. The FOG questionnaire showed a decline of scores after BoNT therapy [78].

Another group was not able to demonstrate any beneficial effect on FOG with 5000 U rimaBoNT/B injected into four areas of the soleus-gastrocnemius complex of the predominantly affected leg, and no significant improvement in nine (BoNT) vs. five (placebo) patients [79].

A good number of patients with PS complain of a restless leg syndrome (RLS). Specific studies in PS, however, are not available. Several small-sized studies were published on the use of BoNT for RLS [80,81,82], including one RCT with 24 patients [83]. 100 U incoBoNT/A were injected into tibialis anterior (40 U), gastrocnemius (40) and biceps femoris muscle (20) on each side. The injection leads to a reduction in severity of symptoms, pain, and improves quality of life (for six weeks), without any adverse effects.

Clinical consequence: At present, the injection of BoNT in the therapy of FOG and RLS in PS cannot be recommended. Further studies are required.

### 3.5. Pain

Pain is a common complaint in PD, with their causes being quite diverse [6,84,85]. This means then that a therapeutic effect can be either direct or indirect. A positive effect of BoNT on pain can be seen by many symptoms, for example, in cervical dystonia, focal dystonia, RLS [84]. Here we refer to the corresponding sections.

A positive effect can be assumed particularly in cases of dystonia [84]. In an uncontrolled study, Pacchetti et al. treated thirty PD patients (22 men and eight women) with “off” painful dystonia with BoNT (see Section 3.3). They injected into tibialis posterior, tibialis anterior, gastrocnemius, flexor digitorum longus, and extensores hallucis longus. In all patients, the pain improved within 10 days, and in 21 patients, the pain disappeared completely for four months [70].

In a study at the Toronto Western Hospital, the indication for therapy with BoNT was examined retrospectively. In the 117 PD patients pain was the main indication for BoNT treatment (50.6%), with dystonic pain in 77.6% and musculoskeletal pain in 22.4% of the cases. Considering pain as indication, 81% of all patients with PD reported benefits after the first BoNT injections, with 53.4% of the cases describing their response as very much improved [85].

In a controlled study, the same group studied the effect of BoNT on limb pain in 12 advanced PD patients [86]. Treatment with BoNT/A showed a significant reduction in numeric rating scale four weeks after the injections, but there was no significant difference compared to placebo.

Clinical consequence: In many indications for BoNT, pain is improved. At present the effect on approval is limited, there is thus no primary indication for BoNT in the treatment of pain in PS.

### 3.6. Focal Hyperhidrosis

Disturbance in perspiration is frequent in PS [87,88], occurring usually profusely and as sudden attacks, whereas focal hyperhidrosis occurs only in rare individual cases [87,88]. In such cases, a focal application of BoNT can be considered. The same holds for an essential hyperhidrosis as well [3].

Clinical consequence: BoNT is only indicated in the therapy for pronounced, focal hyperhidrosis. There is no indication for generalized profuse perspiration.

### 3.7. Gastrointestinal Tract (GIT)

In PD patients, the entire motility of the gastrointestinal tract is disturbed [89]. This leads to sialorrhea in the upper GIT due to reduced swallowing as well as to dysphagia with severe consequences all the way up to aspiration pneumonia [89].

The most frequent use of BoNT for the GIT entails sialorrhea (see Section 2.3). A further area of application is the injection into the upper and lower oesophageal sphincter [90,91,92]. The injection of BoNT is well established for cases of achalasia [93], although specific studies on PS are lacking, as only smaller samples of single case studies have been published. In an early study four patients were treated with 30 U aboBoNT/A in the pre-cricopharyngeal muscle. All four noted improvement up to 16–20 weeks [92]. The dysfunction in the upper esophageal sphincter seems to be more frequent in APS than PD [90]. With insufficient relaxation in the lower esophageal sphincter (LES) an injection is performed into four quadrants of the LES analogous to the therapy for achalasia analog [90].

Alfonsi et al. [90] treated seven PD patients with 15 U onaBoNT/A into the cricopharyngeal muscle, two with improvement. Six of nine PSP, and four of four MSA patients improved. In an open-label study the same research group treated a collective of 67 patients, 21 of whom had PS [91]. All were treated with 15–20 U incoBoNT/A into the cricopharyngeal muscle. Only in the PS patients did they observe a reduction in the percentage of high responders as compared with the first treatment.

An additional gastrointestinal option might involve injection of BoNT into the pyloric sphincter in PD patients with gastroparesis. Up to the present, information on two patients has been published, detailing the injection of 100 U onaBoNT/A, whereby an improved gastric emptying was obtained [94].

There have long been descriptions for the therapeutic effects of BoNT on an anism which caused constipation [95]. Two studies on treatment of constipation were published [96,97]. Here relatively high doses (100 U onaBoNT/A) were injected into the puborectal muscle. Here we should note that anism or muscular outlet obstruction occurs but rarely in PS and seldom constitutes the cause of constipation [98]. PD presents rather more often with slow transit constipation [89].

Clinical consequence: In cases of sialorrhea resulting from swallowing disturbance, the injection of BoNT into the salivary glands is the therapy of choice. In individual cases, an injection with BoNT can be considered for dysfunction of the esophageal and anal sphincters.

## 4. Conclusions

Parkinson’s syndrome (PS) is a multisystem degeneration with a number of motor and non-motor symptoms that have but an insufficient response to dopaminergic medication. In particular, the therapeutic possibilities for the non-motor disturbances are limited and frequently have strong side effects. In some indications, BoNT has demonstrated the capability for closing the gap in treating Parkinson symptoms and to provide symptomatic relief. Thus, BoNT has been approved in the meantime for the treatment of sialorrhea and an overactive bladder. But there are also motor symptoms for which approval has been granted and which occur frequently in PD, for example cervical dystonia and blepharospasm, which are frequently overlooked in PS and are thus not subject to treatment trials. A number of other indications are possible or should be studied further, e.g., tremor, and focal dystonia. The therapeutic possibilities of BoNT for treating symptoms in PS have not yet been exhausted. The same symptoms are to be discussed in the case of APD. In PSP, neck rigidity and blepharospasm/lid opening inhibition, in MSA Anterocollis, blepharospasm/lid opening inhibition and sialorrhea and in CBS focal dystonia should be mentioned in particular.

## Figures and Tables

**Table 1:** Indications for botulinum neurotoxin in Parkinson’s disease (PD) (recommendations according the level of evidence is not meaningful because of limited data). * Not specified for PD.

	Motor	Non-Motor
Approved or licensed	Cervical dystonia * Blepharospasm *	Sialorrhea Overactive bladder
Probably or possibly effective	Lid opening inhibition Tremor Focal dystonia -hand and foot -laryngeal -oromandibular	Dysphagia Outlet constipation Focal hyperhidrosis
Conflicting data	Camptocormia Pisa syndrome	

## Data Availability

Not applicable.

## Abbreviations

BoNT	Botulinum Neurotoxin
aboBoNT/A	AbobotulinumtoxinA
incoBoNT/A	IncobotulinumtoxinA
onaBoNT/A	OnabotulinumtoxinA
rimaBoNT/B	RimabotulinumtoxinB
U	Units
PD	Parkinson’s disease
PS	Parkinson’s syndrome
APS	Atypical Parkinson’s syndrome
EMG	Electromyography
RLS	Restless leg syndrome
SPC	Summary of Product Characteristics
